# Candidate Cell Substrates, Vaccine Production, and Transmissible Spongiform Encephalopathies

**DOI:** 10.3201/eid1712.110607

**Published:** 2011-12

**Authors:** Pedro Piccardo, Larisa Cervenakova, Irina Vasilyeva, Oksana Yakovleva, Igor Bacik, Juraj Cervenak, Carroll McKenzie, Lubica Kurillova, Luisa Gregori, Kitty Pomeroy, David M. Asher

**Affiliations:** University of Edinburgh, Easter Bush, UK, (P. Piccardo);; Food and Drug Administration, Kensington, Maryland, USA (P. Piccardo, I. Bacik, J. Cervenak, L. Kurillova, L. Gregori, K. Pomeroy, D.M. Asher);; Holland Laboratory American Red Cross, Rockville, Maryland, USA (L. Cervenakova, I. Vasilyeva, O. Yakovleva, C. McKenzie)

**Keywords:** prion, prion protein, BSE, bovine spongiform encephalopathy, Creutzfeldt-Jakob disease, CJD, vaccines, TSE, transmissible spongiform encepthalopathy

## Abstract

Transmissible spongiform encephalopathy (TSE) agents have contaminated human tissue–derived medical products, human blood components, and animal vaccines. The objective of this study was to determine the potential susceptibility to infection of 5 cell lines used or proposed for manufacture of biological products, as well as other lines. Cell lines were exposed to the infectious agents of sporadic and variant Creutzfeldt-Jakob disease and bovine spongiform encephalopathy (BSE). Exposed cultures were tested for TSE-associated prion protein (PrP^TSE^) and TSE infectivity by assay in rodents and nonhuman primates. No PrP^TSE^ or infectivity has been detected in any exposed cell line under study so far. Animals inoculated with BSE brain homogenate developed typical spongiform encephalopathy. In contrast, animals inoculated with cells exposed to the BSE agent remained asymptomatic. All cell lines we studied resisted infection with 3 TSE agents, including the BSE agent.

## Abstract 2

Candidate cell substrates neither accumulated abnormal prion protein nor propagated infectivity.

Transmissible spongiform encephalopathies (TSEs or prion diseases) are a heterogeneous group of fatal neurodegenerative diseases that affect animals and humans. TSEs can be sporadic, transmitted iatrogenically, or expressed as familial disorders. TSEs include scrapie in sheep and goats, bovine spongiform encephalopathy (BSE) in cattle, chronic wasting disease in cervid ruminants, and mink encephalopathy. In humans the most common TSEs are sporadic, familial, and variant Creutzfeldt-Jakob disease (sCJD, fCJD, and vCJD, respectively). TSEs are characterized by the accumulation in the central nervous system and, less often, in lymphoid tissues of TSE-associated prion protein (PrP^TSE^), a conformational variant of a normal host cellular prion protein (PrP^C^). PrP^C^ is a nonessential protein but, at least in mice and cows, must be expressed by animals susceptible to TSE infection. There is compelling evidence that the BSE agent has infected humans, causing vCJD. Most cases of vCJD are attributed to exposure to contaminated beef products (*1**–**3*). In addition, vCJD infections have been transmitted by transfusions of nonleukoreduced erythrocyte concentrates and by a human-derived coagulation factor (factor VIII) (*4**–**6*).

The conclusion that PrP^TSE^ is central to the pathogenesis of TSE is based on the temporal and anatomic correlations between accumulation of PrP^TSE^ and the development of pathologic changes in tissues of the central nervous system (*1*). However, TSEs can develop in the absence of detectable PrP^TSE^ and, conversely, PrP^TSE^ might accumulate without causing either clinical illness or the neuropathologic alterations typical of TSEs (i.e., a progressive fatal illness with spongiform degeneration of the brain) (*7**,**8*). In short, the molecular basis of TSE infection and the role of PrP^TSE^ (unquestionably important in pathogenesis of TSEs) are not yet entirely clear, and both remain key issues in TSE research (*9**,**10*). The standard assay for detecting a TSE agent remains bioassay in susceptible animals.

Many investigators once believed that TSE agents infected mainly, if not exclusively, cells of neuronal and lymphoid lineages. It has become clear, however, that the susceptibility of cells to infection with TSE agents cannot be reliably predicted either from their tissue of origin or level of expression of PrP^C^ (*11**,**12*). Studies showing that murine fibroblast cell lines are susceptible to infection with mouse-adapted scrapie agent (*11**,**12*) increased concern that nonneuronal cell substrates used to propagate viruses for vaccine production might become infected with a TSE agent contaminating some component of culture medium, especially bovine serum (*13*). The theoretical risk of contaminating vaccines or other biologic products prepared in culture cells with TSE agents from animal-derived materials in media has been considered low. However, 1) as noted above, the blood of asymptomatic humans has transmitted vCJD, and 2) in a variety of experimentally TSE-infected animals, TSE agent has been detected in blood, mainly in nucleated cells and plasma (*4**–**6**,**14**–**17*). Fortunately, no human vaccine has ever been implicated as a source of iatrogenic TSE. However, 2 animal tissue–derived vaccines have caused outbreaks of scrapie in sheep, and 2 medical products of human origin—dura mater allograft and human cadaveric pituitary hormones (no longer marketed in the United States)—have transmitted hundreds of cases of CJD; corneal grafts have transmitted a few cases as well (*2**,**18*). Since 1993, the US Food and Drug Administration has recommended against the use in the manufacture of biological products of bovine-derived materials from countries identified by the US Department of Agriculture (USDA) as having BSE or being at increased risk for BSE in native cattle (*19*).

The recognition of >20 BSE cases in North America since 2003 (most in Canada) has increased the need to determine whether cell substrates that might be accidentally exposed to the BSE agent are capable of acquiring and propagating the infectious agent and potentially transmitting infections to vaccine recipients (*20*). To address these issues, we investigated the susceptibility of cell lines used or proposed for manufacture of biologics and controls to propagate TSE agents, especially the BSE agent, under simulated worst-case conditions.

## Materials and Methods

### Cell Cultures Exposed to TSE Agents

We studied the following 5 actual or candidate cell substrates used or proposed for production of biologic products: CHO-K1 (Chinese hamster ovary ATCC-CCL61); Vero C1008 (African green monkey kidney ATCC-CRL1586); WI-38 (human lung diploid fibroblasts ATCC-CCL75); MDCK (dog kidney ATCC-CCL34); and HEK-293 (human embryonic kidney ATCC-CRL-1573 transformed with defective adenovirus as a surrogate for PER.C6) (Crucell, Leiden, Netherlands) (Table 1).

**Table 1:** Cell cultures exposed to transmissible spongiform encephalopathy agents and propagated for 30 passages*

Variable	sCJD	vCJD	BSE	22L-scrapie
Actual or candidate cell lines for vaccine production				
CHO-K1	Y	Y	Y	
Vero C1008	Y	Y	Y	
WI-38	Y	Y	Y	
MDCK	Y	Y	Y	
HEK-293	Y	Y	Y	
Other cells resistant to TSE infection				
MBDK			Y	
EBTR			Y	
BT			Y	
BCE C/D-1b			Y	
BL3.1			Y	
R9ab	Y	Y	Y	
Cells infectable with 22L scrapie				
Mo3F4-3T3				Y
L929				Y

*sCJD, sporadic Creutzfeldt-Jakob disease; vCJD, variant Creutzfeldt-Jakob disease; BSE, bovine spongiform encephalopathy; CHO-KI, Chinese hamster ovary; Vero C1008, African green monkey kidney; WI-38, human lung diploid fibroblasts; MDCK, dog kidney; HEK-293, human embryonic kidney; MBDK, bovine kidney; EBTR, bovine trachea; BT, bovine turbinate; BCE C/D-1b, bovine cornea; BL3.1, bovine B lymphocytes; R9ab, rabbit fibroblasts; Mo3F4-3T3, mouse embryo fibroblasts; L929, mouse embryo fibroblasts; Y, experiment was completed.

We used bovine-derived cell lines MBDK (bovine kidney, ATCC-CCL22); EBTR (bovine trachea, ATCC-CCL44); BT (bovine turbinate, ATCC-CRL1390); BCE C/D-1b (bovine cornea, ATCC-CRL2048); and BL3.1 (bovine B lymphocytes ATCC-CRL 2306) in an attempt to develop a cell culture assay for BSE agent. As a probable negative control, we used R9ab (rabbit fibroblasts ATCC CCL-193) known to resist infection with the scrapie agent. Finally, we used NIH-3T3 (ATCC-CRL1658) and L929 (ATCC-CCL1), both mouse embryo fibroblast cells, known to be infectable with the mouse-adapted 22L strain of scrapie agent as positive controls (*11*) (Table 1).

### TSE Agents

BSE, vCJD, and sCJD agent inocula were 1% suspensions (wt/vol). Controls were similar uninfected brain suspensions. CJD-infected human brain suspensions were World Health Organization (WHO) Candidate Biologic Reference CJD Materials prepared in 0.3 mmol/L sucrose (*21*). The infectivity titer for vCJD (WHO 98–145) in transgenic mice expressing the human PrP gene (TgHu) was 6.1 log_10_ intracerebral inoculation (ic) with 50% infectious dose (ID_50_) per 30 μL; sCJD (WHO 97–008) was 5.4 log_10_ ic with ID_50_/30 μL; and sCJD (WHO 99–009) was 6.1 log_10_ ic ID_50_/30 μL (L. Cervenakova, unpub. data).

The BSE-infected bovine brain was a 10% brain suspension in 250 mmol/L sucrose. The infectivity titer of that material is being determined in squirrel monkeys (>10^2^/inoculum [*22*]) intracerebral 150 μL and intraperitoneally 150 μL with 10^−1^ (wt/vol) through 10^−9^ dilutions of low speed–clarified brain extracts. Nonhuman primates were also inoculated with a 10^−2^ dilution of brain extract after filtration through a 0.45-μm Millipore membrane to eliminate bacterial contamination. Three animals each were inoculated with the BSE agent dilution 10^−1^ to 10^−6^; 2 animals each were inoculated with the same material dilution 10^−7^ to 10^−9^. Similar bacteria-free samples were used to expose cell substrates. The ic infectivity titer of the BSE agent in transgenic mice expressing the bovine PrP gene (TgBo) was 5.0 log_10_ LD_50_/30 µL (L. Cervenakova, unpub. data). Brain extracts from animals with neurologic signs contained PrP^TSE^ by Western blot (WB) analysis, and TSE was confirmed neuropathologically. All BSE experiments were performed under BioSafety Level 3 containment conditions in facilities inspected and approved by the USDA. Scrapie agent used as a positive control was the 22L mouse-adapted strain (*11*).

### Inoculation of Cells with TSE Agents

Cells were grown in 25-cm^2^ tissue culture flasks and overlaid with 200 µL of a detergent-free homogenate of brain tissue diluted to 1% (wt/vol) in Opti-MEM (Gibco, Grand Island, NY, USA). Bacteria-free inocula were obtained by filtration of 1% brain extracts through premoistened 0.45-μm Millipore membranes (Millipore, Billerica, MA, USA). In an attempt to mimic a worst-case scenario, in some instances cells were exposed to brain extracts by gently centrifuging them in 2- cm^2^ tissue culture flasks for 5 min at ≈900 × *g* to ensure maximum contact with TSE agents. After incubation of the cell cultures at 37°C for 4 h, 400 µL of fetal bovine serum/Dulbecco modified Eagle medium was added, and cells were incubated for 96 h before further passaging. To determine the presence of PrP^TSE^ in cells, WB analyses were done, usually at passages 0, 5, 10, 15, 20, and 30. The same protocol was used with NIH-3T3 and L929 murine cells exposed to mouse-adapted 22L scrapie agent (as positive controls).

### Animal Models

TgHu and TgBo mice were developed at the American Red Cross (L. Cervenakova, unpub. data). TgHu mice express in the brain ≈4-fold higher levels of PrP than wild-type mice. TgBo mice express in the brain wild-type levels of PrP. These Tg mice do not develop spontaneous neurologic illness. Neuropathologic studies of selected aged mice showed no abnormalities such as vacuolation of the neuropil typical of TSEs. Conventional C57/BL6 mice were used to titrate 22L scrapie agent. Squirrel monkeys were purchased from Osage Research Primates (Kaiser, MO, USA) and housed in a USDA-approved BioSafety Level 3 animal facility. In a separate series of experiments, a work in progress, we are addressing the hypothesis, postulated by some authorities (*13*), that a TSE agent might develop spontaneously in cell cultures expressing mutated or nonmutated PrP. To that end, we are investigating whether transfected cell lines overexpressing wild-type or mutant PrP might become spontaneously infectious. Transfected cells are being bioassayed in squirrel monkeys. Although none of those monkeys showed development of any neurologic disease (7 years after inoculation), 3 animals have died of unrelated causes (1 with pneumonia and 2 culled with unexplained nontuberculous granulomatosis) without evidence of TSE neuropathologic changes. We used tissues of those 3 monkeys as negative controls. All animal experiments were reviewed and approved by the Food and Drug Administration and the American Red Cross Institutional Animal Care and Use Committees.

### Preparation of Inocula for Bioassay in Mice and Primates

Samples of selected cultures at various passage levels and the final subcultures after >30 passages were examined for PrP^TSE^ by WB by using published protocols (*14*). Tg mice were inoculated intracerebrally with 30 μL of cell lysates from 1 × 10^8^ cells/mL to 2 × 10^9^ cells/mL (depending on the cell line) by 3 cycles of freezing and thawing followed either by sonication or by forcing through hypodermic needles of increasingly small gauge. Tg mice were inspected daily for signs of illness and euthanized either after illness developed or at the end of a normal expected lifespan (Table 2, Table 3). The same neuropathologist, blinded to the experimental design, interpreted all stained sections used for neuropathologic studies. The same cell lysates were also inoculated into primates, 150 μL intracerebrally and 150 μL intraperitoneally. Animals were inspected daily for signs of illness and promptly euthanized when definite neurologic signs or intercurrent illnesses developed. Animals without clinical illness will be maintained for their expected normal lifespans. After euthanasia, brains were removed and portions stored frozen for WB (*22*) or in 10% formalin solution for histopathologic and immunohistochemical studies by using published protocols (*8*).

**Table 2:** Cell lines exposed to BSE or vCJD or normal bovine or human brain suspension and bioassayed in TgBo mice*

Cell line	No. mice	dpi	No. mice	dpi

BSE	NB	vCJD	NB
CHO	21	9	720	17	9	730
Vero	20	10	730	ND	ND	ND
WI-38	23	10	730	ND	ND	ND
R9ab	18	9	630	ND	ND	ND
MDCK	19	10	730	20	10	730
HEK-293	20	20	730	ND	ND	ND
Mo3F4-3T3	17	10	750	ND	ND	ND

*BSE, bovine spongiform encephalopathy; vCJD, variant Creutzfeldt-Jakob disease; NB, normal brain; dpi, days postinculation at cull; ND, not done; CHO, Chinese hamster ovary; Vero, African green monkey kidney; WI-38, human lung diploid fibroblasts; R9ab, rabbit fibroblasts; MDCK, dog kidney; HEK-293, human embryonic kidney; Mo3F4-3T3, mouse fibroblasts.

**Table 3:** Cell lines exposed to sCJD or normal human brain suspension and bioassayed in TgHu mice*

Cell line	No. mice	dpi

sCJD	NB
Vero	19	10	650
CHO	18	9	580
R9ab	18	10	580

*sCJD, sporadic Creutzfeldt-Jakob disease; TgHu, transgenic mice expressing human prion protein gene; NB, normal brain; dpi, days postinoculation at cull; Vero, African green monkey kidney; CHO, Chinese hamster ovary; R9ab, rabbit fibroblasts.

## Results

### TSE in TgBo Mice and Primates Inoculated with BSE agent

**Figure 1:**
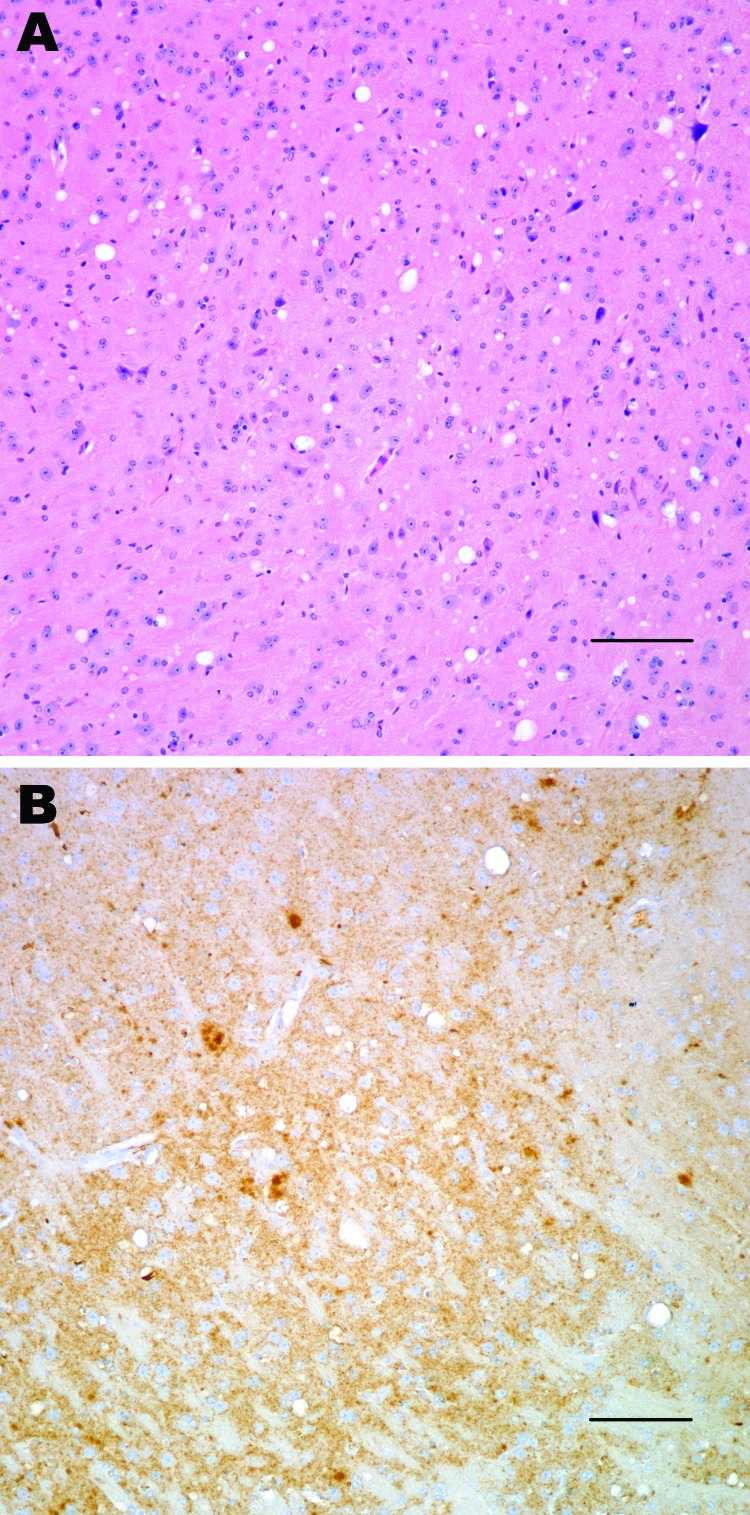
Histopathologic analysis of transgenic mouse expressing bovine prion protein (PrP) gene inoculated with bovine spongiform encephalopathy agent. Spongiform degeneration in the thalamus (A), adjacent section showing PrP immunopositivity (B). Panel A was stained with hematoxylin and eosin, panel B was immunostained with PrP antibody 6D11. Scale bars = 100 μm.

Neuropathologic characterization of TgBo mice inoculated with BSE agent showed spongiform degeneration in the cerebrum with variable amounts of fine-punctate, coarse, and, in some cases, plaque-like deposits in the cerebrum, cerebellum, and brain stem. Mice inoculated with the bacteria-free filtrate of 1% BSE-infected brain suspension used to expose cells also developed signs of TSE, and PrP^TSE^ was detected in brains, demonstrating that the inoculum used to expose cell cultures contained a TSE agent transmissible to mice (Figure 1).

**Figure 2:**
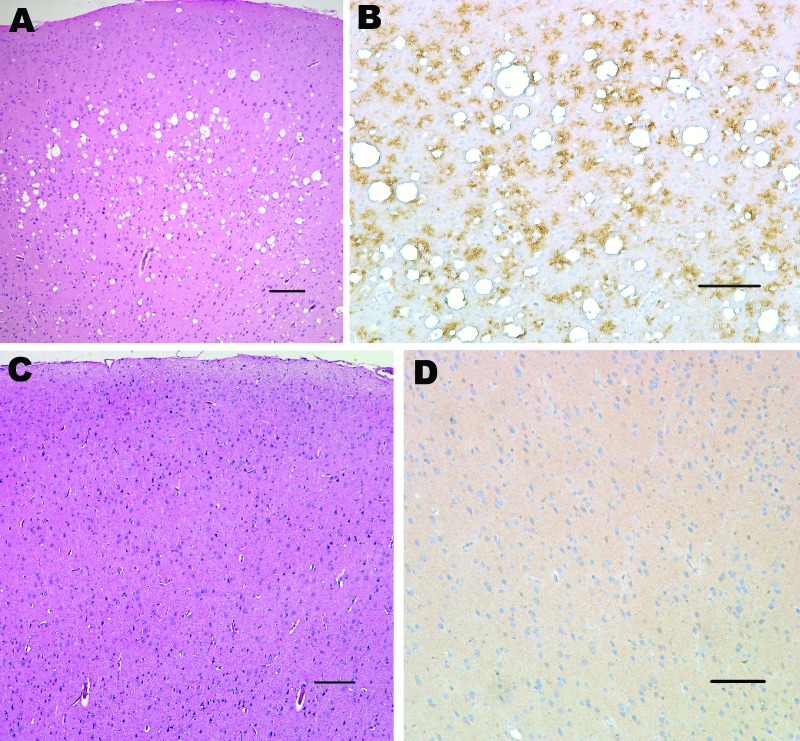
Histopathologic analysis of squirrel monkey inoculated with bovine spongiform encephalopathy agent (A, B). Spongiform degeneration in the cerebral cortex (A), adjacent section showing abundant prion protein (PrP) immunopositivity (B). Squirrel monkey without transmissible spongiform encephalopathy (C, D). Cerebral cortex with no spongiform degeneration (C), absence of PrP positivity in the cerebral cortex (D). Panels A and C correspond to sections stained with hematoxylin and eosin; panels B and D were sections immunostained with PrP antibody 6D11. Scale bars = 150 μm.

Squirrel monkeys were inoculated with serial dilutions of the same material used to inoculate TgBo mice. At the time this report was written, 6 monkeys have already developed neurologic signs typical of TSE. Three animals inoculated with 10^–1^ (10%) unfiltered low-speed clarified BSE reference material became ill and were euthanized ≈3.2 years after inoculation; 2 primates inoculated with 10^–2^ unfiltered, low speed–clarified BSE suspension were euthanized 3.7 years after inoculation; and 1 primate inoculated with the 0.45-μm filtered bacteria-free 10^–2^ (1%) BSE-infected brain suspension used to expose cells also developed signs of TSE and was euthanized 3.3 years after inoculation. Brain extracts from each of these monkeys contained PrP^TSE^ demonstrated by WB. Preliminary neuropathologic studies of formalin-fixed paraffin-embedded brain sections of each of the 6 animals showed severe spongiform degeneration of the cerebrum, cerebellum, and brainstem. Immunohistochemical studies showed widespread accumulations of PrP-immunopositive deposits throughout the brain of each monkey (Figure 2). Control brains from 4 other squirrel monkeys dying of nonneurologic diseases, including 1 housed with monkeys used to titrate BSE agent, showed no evidence of TSE (Figure 2). A detailed neuropathologic report of all monkeys is in press (*22*).

### Attempts to Infect Actual or Candidate Cell Substrates

#### Analysis of Cell Cultures and Bioassay in Rodents

**Figure 3:**
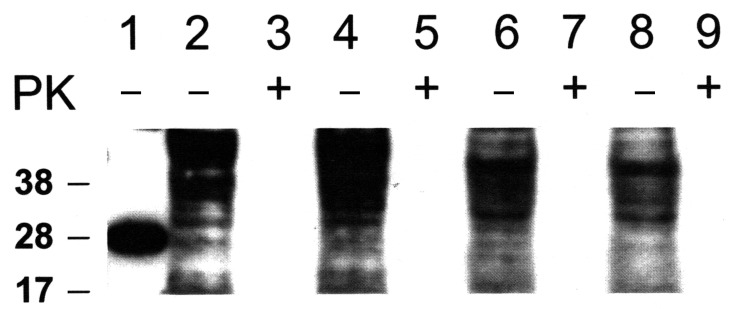
Western blot of recombinant prion protein (PrP) 5 ng (lane 1), CHO cells (lanes 2–5) and Vero cell (lanes 6–9). Cells exposed to normal bovine brain and passaged 30 times (lanes 2, 3, 6, 7). Cells exposed to bovine spongiform encephalopathy agent and passaged 30 times (lanes 4, 5, 8, 9). Total PrP (cell extracts without proteinase K [PK] digestion) are shown in lanes 2, 4, 6, 8; cell extracts treated with PK are shown in lanes 3, 5, 7, 9. Western blots were probed with PrP monoclonal antibody 6D11.

No PrP^TSE^ was detected in cells exposed to normal brain extracts (controls) or after passage 5 in any of the cell substrates or control TSE-resistant cells exposed to brain extracts containing TSE agents. PrP^TSE^ was detected in some samples of cells collected 96 h after inoculation (passage 0), suggesting the probable presence of residual inoculum. The consistent failure to detect PrP^TSE^ in any cell line exposed to TSE agents after >5 passages suggests that proteinase K–resistant PrP was not generated de novo under these experimental conditions. WB analyses of cells collected after 30 serial passages showed no detectable PrP^TSE^ in any cell line (Figure 3). Samples of each cell line exposed to human TSE agents (sCJD, vCJD) and BSE agent were expanded after 30 passages for bioassay in TgHu and TgBo mice. At the time of this report, no mice inoculated with cells exposed to TSE agent have evidence of TSE illness during their expected lifespan (Table 2, Table 3). WB with extracts of brain tissue from all culled animals showed no PrP^TSE^. Neuropathologic analyses of selected mouse brains found no spongiform encephalopathy or accumulations of PrP (data not shown). The results confirm that no cell substrate propagated infectivity detectable by mouse bioassay, even when mice were observed for their expected lifespan.

#### Nonhuman Primate Bioassay

Lysates of Vero, CHO, WI-38, and R9ab cells exposed to BSE agent and passaged 30 times were inoculated into squirrel monkeys in May 2006. Lysates of MDCK and HEK-293 cells exposed to BSE agent and passaged 30 times were inoculated into squirrel monkeys in August 2007. No monkey inoculated with those cells had neurologic signs. One monkey inoculated with Vero cells exposed to the BSE agent was attacked by a cage mate; the wound became infected, suppuration increased despite treatment with antimicrobial drugs, and the injured monkey was euthanized several days later without ever showing any sign of neurologic disease. The brain showed no neuropathologic changes of TSE or PrP^TSE^ by WB (data not shown). In contrast, as noted above, primates inoculated with Swiss Reference BSE brain extracts at 10^–1^ and 10^–2^ dilutions developed typical TSE confirmed by neuropathologic results (Figure 2) and by WB (not shown). These results indicate that 1) squirrel monkeys are susceptible to infection with the BSE agent, and 2) BSE infectivity was present in the bacteria-free filtrate used to expose cell substrates.

### Murine Fibroblast Cell lines Generate PrP^TSE^ after Exposure to Mouse-adapted Scrapie Agent

**Figure 4:**
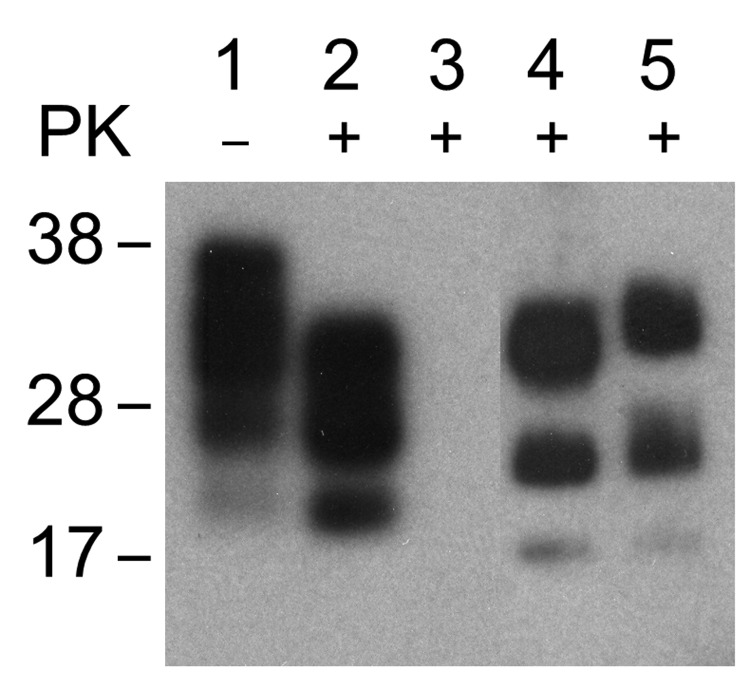
Western blot of brain extract from C57/Bl mouse inoculated with 22L mouse-adapted scrapie agent (lanes 1, 2); NIH-3T3 cells exposed to normal mouse brain and passaged 30 times (lane 3); NIH-3T3 (lane 4) and L929 (lane 5) cells exposed to 22L scrapie agent and passaged 30 times. Nonproteinase K [PK]–treated samples (lane 1), PK-treated samples (lanes 2–5). Western blots were probed with prion protein monoclonal antibody 6H4.

Several murine tissue cultures have been successfully infected with TSE agents, providing a promising alternative to assays of TSE agents by time-consuming and expensive bioassays in animals (*9**,**11**,**12**,**23**–**28*). Previous studies reported that several commonly used mouse fibroblast cell lines can be efficiently infected with the scrapie agent and support formation of PrP^TSE^ (*11*). We studied 2 such cell lines as a positive control to confirm that our protocol would have detected an infected cell line and that our failures to find PrP^TSE^ or infectivity by bioassay in cell substrates exposed to the BSE agent were more likely to have resulted from an intrinsic resistance of the cells to infection rather than to some technical problem. We exposed monolayers of NIH-3T3 and L929 murine fibroblast cells to the mouse-adapted 22L strain of scrapie agent and observed the formation of readily detectable PrP^TSE^ that persisted through 30 passages (Figure 4). We are performing bioassays of the PrP^TSE^-positive cells in C57/BL6 mice to determine amounts of infectivity. Several mice inoculated with samples of NIH-3T3 and L929 fibroblasts collected 30 passages after exposure to 22L mouse-adapted scrapie agent have already developed spongiform encephalopathy, confirming that the agent was successfully propagated in vitro (unpub. data).

## Discussion

Candidate cell substrates used to produce biologics were not infected by a simulated worst-case exposure to BSE agent. Similar more limited studies exposing the same cultures to vCJD and sCJD agents also gave negative results.

The finding of PrP^TSE^ in several cell culture samples collected at passages 0–4 probably resulted from small amounts of residual inoculum. This conclusion is reinforced by our consistent failure to detect PrP^TSE^ in any cell sample at or after passage 5. However, we cannot rule out the possibility of a transient de novo generation of PrP^TSE^ in the earliest passages of the cultures. Other investigators (*23*) have shown some immediate (acute) formation of new PrP^TSE^ in infection-resistant cell cultures exposed to scrapie agent; the new PrP formed did not depend upon the strain of TSE agent used or cell type involved and was not associated with infectivity (*23*). Whether the failure of infectivity to persist after transient formation of PrP^TSE^ resulted from death of the infected cells or the dilution of a small amount of TSE agent is unknown. We recognized no overt cytotoxicity in any cell line inoculated with a TSE-infected brain suspension. Thus, our data so far suggest that several cell substrates actually or potentially used to produce biologics were not susceptible to the propagation of TSE agents under the experimental conditions we used. In agreement with these results, others showed that MDCK cells were refractory to infection with human and mouse TSE agents (*24*). MRC5 human diploid cells also failed to support the replication of a TSE agent (*25*).

Because bioassays are time-consuming and expensive, a few lines of cells susceptible to infection with certain strains of TSE agent have been derived (*9**,**11**,**12**,**26**–**30*). However, for unknown reasons, most cell lines have resisted TSE infections. In addition, most cell lines infectable with TSE agents have been highly heterogeneous and not stable, requiring repeated subcloning of susceptible cells, and they have been successfully infected with only a few strains of TSE agent (*27*). Thus, it was vital to verify that the protocol we chose as a simulated worst-case model would successfully infect previously characterized cell lines with a TSE agent to which they were known to be susceptible. We demonstrated that the protocol we used was valid by persistently infecting 2 murine fibroblast cell lines with a mouse-adapted scrapie agent. However, we must caution that even cell lines susceptible to infection have shown widely different responses when exposed to various TSE agent strains (*27*). Furthermore, the emergence of atypical forms of BSE raises a new concern, i.e., that cell substrates resistant to infection with the original classic BSE agent (*31*) might not resist infection with newer strains of BSE agent (if new strains are implicated in atypical BSE).

Although BSE has been transmitted to many animal species, the efficiency of transmission between species has been difficult to predict. Low transmission rates and long incubation periods are often observed when TSEs are transmitted to a new species—commonly known as a species barrier (*32*)—but experience with squirrel monkeys (sensitive to almost all TSE agents affecting humans and to several animal TSEs as well) suggests that they are an especially useful model species (*33*). To that end we initiated titration experiments of the BSE agent and found at the time of writing this report that squirrel monkeys develop TSE when inoculated with BSE brain suspensions at high concentration 10^–1^ and 10^–2^ dilutions (work in progress). Given the long incubation time (years) needed to elicit disease in this primate model, we will continue the observation of monkeys inoculated with cell cultures exposed to the BSE agent in an attempt to determine whether low infectivity could potentially be detected in this model.

Because few cell lines used as substrates for biologics are of bovine origin, it is tempting to speculate that the potential for contaminating biologics with the BSE agent is low. However, an observation that cells derived from a pheochromocytoma of the rat adrenal medulla (PC12 cells) could be infected with a mouse-adapted scrapie agent and murine hypothalamic cells with a human TSE agent demonstrates that cross-species transmissions of TSE agents in cells in culture are possible (*29*). Recent studies showed that passage of TSE agents through different animal species altered key characteristics of the agent, sometimes producing variants with increased virulence or broader host range, as happened when BSE agent was passaged through sheep (*34*). Thus, further experiments are needed to evaluate the potential susceptibility of various cultured cell lines to infection with new emerging TSE strains.
